# Perspectives of women and healthcare providers on facilitators and barriers to cervical cancer screening in the rural Kilimanjaro region Tanzania: a qualitative study

**DOI:** 10.1080/16549716.2026.2735552

**Published:** 2026-09-28

**Authors:** Rogathe F. Machange, Bernard Njau, Christina C. Mtuya, Victor W. Katiti, Lyidia Masika, Bariki Mchome, Eva Kantelhardt, Mirgissa Kaba, Blandina T. Mmbaga, Declare L. Mushi, Gunilla Björling, Rachel Manongi

**Affiliations:** a School of Public Health, KCMC University, Moshi, Tanzania; b Kilimanjaro Christian Medical Centre, Moshi, Tanzania; c Department of Research, Kilimanjaro Clinical Research Institute, Moshi, Tanzania; d Department of Nursing, Kilimanjaro Christian Medical Centre; e Department of Obstetric and Gynaecology, Kilimanjaro Christian Medical Centre; f Department of Gynecology, Martin-Luther-University Halle-Wittenberg, Halle, Germany; g School of Public Health, Addis Ababa University, Ethiopia; h Department of Paediatric, Kilimanjaro Christian Medical Centre; i Department of Neurobiology Care Sciences and Society, Karolinska Institutet, Stockholm, Sweden; j School of Health and Welfare, Jönköping University, Jönköping, Sweden; k Södertälje Hospital, Division of Anaesthesia, Operation, and Intensive Care, Södertälje Hospital, Södertälje, Sweden

**Keywords:** Cervical cancer, behavior, screening, community, TDF/COM-B

## Abstract

**Background:**

Cervical cancer can be prevented and treated if detected early through regular screening. However, 88% of women aged 30 to 49 have never been screened in Tanzania.

**Objective:**

This study explored facilitators and barriers to cervical cancer screening among women and healthcare providers in rural Kilimanjaro, Tanzania, using the Theoretical Domains Framework and the Capability, Opportunity, Motivation-Behavior model.

**Methods:**

An exploratory qualitative design was employed; data were collected from March to October 2024. Six focus group discussions were conducted with 54 women aged 30–50 years, and twelve in-depth individual interviews with healthcare providers. Data were transcribed, coded, and thematically analyzed using QDA Miner Lite v2.0.8.

**Results:**

Four main themes emerged, each with sub-themes: (1) knowledge about cervical cancer and screening (disparities in knowledge between screened and non-screened women; healthcare providers’ knowledge and training needs; age and parity as knowledge mediators); (2) power of social influence (social facilitation; social barriers); (3) emotional and structural influences on cervical cancer screening (emotional barriers and coping; structural and logistical barriers); and (4) enhancing cervical cancer screening uptake (targeted community education and outreach; improving service accessibility). Barriers include limited knowledge, misconceptions, fear of pain and positive results, limited access to services, and a shortage of trained healthcare providers. Social support from families, peers, community leaders, and healthcare providers was identified as a strong facilitator.

**Conclusion:**

Increasing cervical cancer screening uptake in rural communities requires targeted, theory-informed interventions that incorporate education to enhance awareness, strengthen social support, and improve health services.

## Paper Context

- **Main findings**: Four themes with eight sub-themes were identified. Barriers include limited knowledge and misconceptions, fear of pain and positive results, and limited access and staff. Facilitators include social support from families, peers, and community leaders, and targeted community education and outreach.
- **Added knowledge**: Highlights the need for context-specific, theory-informed interventions integrating community education, social support, and health system strengthening to improve CC screening in low-resource settings.
- **Global health impact for policy and action**: Provides baseline evidence from low-resource settings to inform targeted strategies for increasing CC screening coverage toward the WHO 70% screening target.

## Background

Cervical cancer (CC) is one of the most preventable and curable forms of cancer, especially when detected and treated early. Despite this, it remains a major public health concern and is the fourth most common cancer among women globally [1]. Each year, approximately 660,000 new cases and 350,000 deaths occur, with 94% of deaths happening in low- and middle-income countries. In sub-Saharan Africa, CC is the second most common cancer-causing death in women [2]. In Tanzania, CC is the leading cancer among women aged 15–44, with an incidence rate of 9,770 and a mortality rate of 6,695 per 100,000 women [3].

The World Health Organization (WHO) launched a global strategy in 2020 to eliminate CC. The strategy includes screening 70% of women aged 30–49 at least twice in their lifetime and ensuring 90% of women with precancerous lesions receive treatment by 2030 [3,4]. However, Tanzania still faces significant challenges in achieving this target. Despite offering free screening services since 2004 [3], only 12% of eligible women had been screened by 2021–2022, highlighting significant limitations in the national screening program implementation. While women’s perceptions have been widely studied, healthcare providers play a pivotal role in influencing access, quality, and utilization of screening services [5]. To increase screening uptake, it’s crucial to understand facilitators and address barriers to CC screening to both women and healthcare providers [6].

Moreover, for effective implementation of CC screening, a mutual collaboration between women, health care providers, religious, government leaders and other stakeholders probably needs to be addressed. Health care providers play a central role in the provision of CC screening services for the community to accept the available health services.

Early screening is a crucial strategy for preventing CC [7]. However, uptake of CC screening among women is low partly due to factors like limited knowledge, fear, privacy concerns, and cultural barriers [8–10]. Facilitators such as supportive attitudes, education, and trusted environments have been reported to increase CC screening uptake [11–14]. This study used the TDF and COM-B frameworks to systematically identify behavioral barriers and facilitators, as they provide a comprehensive, integrated, and implementation-oriented approach to understanding behavior and informing targeted behavior change interventions [15–17]. The TDF integrates 33 behavioral theories into 14 domains to identify cognitive, social, and environmental influences on behavior as seen in Table 1 [16,19]; while the COM-B model explains behavior as the result of the interaction between capability, opportunity, and motivation. Capability includes psychological and physical capacity, Opportunity encompasses environmental and social factors, and Motivation involves reflective and automatic processes; all three components are essential for effective behavior change [18] (see Figure 1). These frameworks help tailor interventions to the community (see Table 2).

**Figure 1.**
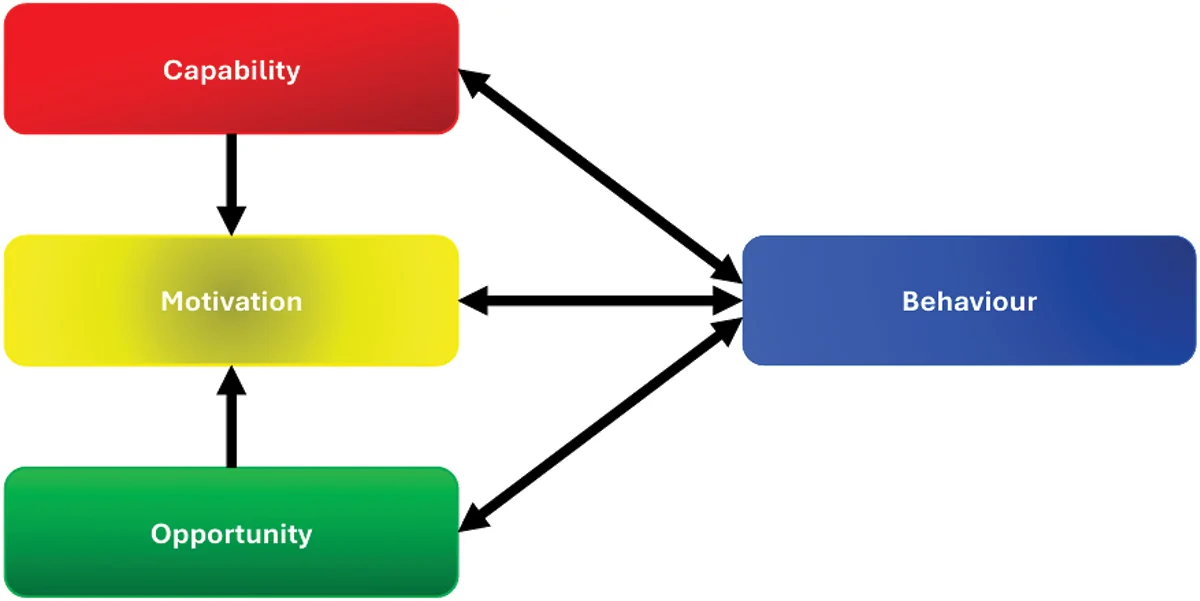
The COM-B model.

*Note: Adapted from Buchanan et al. (2012)* [18].

**Table 1.** The theoretical domains framework.

TDF Domain Knowledge	Definition
Knowledge	Information relating to the behavior
Skills	An ability or proficiency acquired through practice
Social/Professional Role and Identity	A coherent set of behaviors and displayed personal qualities of an individual in a social or work setting
Beliefs about Capabilities	Acceptance of the truth, reality or validity about an ability, talent or facility
Optimism	The confidence that things will happen for the best or that desired goals will be attained
Beliefs about Consequences	Acceptance of the truth, reality or validity about outcomes of a behavior in a given situation
Reinforcement	Increasing the probability of a response by arranging a dependent relationship, or contingency, between the response and a given stimulus
Intentions	A conscious decision to perform a behavior or a resolve to act in a certain way
Goals	Mental representations of outcomes or end states that an individual wants to achieve
Memory, Attention and Decision Processes	The ability to retain information, focus selectively on aspects of the environment and choose between two or more alternatives
Environmental Context and Resources	Any circumstance of a person’s situation or environment that discourages or encourages the development of skills and abilities, independence, social competence and adaptive behavior
Social influences	Those interpersonal processes that can cause individuals to change their thoughts, feelings or behaviors
Emotion	A complex reaction pattern, involving experiential, behavioral, and physiological elements, by which the individual attempts to deal with a personally significant matter or event
Behavioral Regulation	Anything aimed at managing or changing objectively observed or measured actions

Note: Adapted from Buchanan et al. (2012) [18].

**Table 2.** The COM- B model and its relation to the TDF.

COM- B component		TDF domain
Capability	Psychological	Knowledge Skills Memory, Attention and Decision Processes Behavioral regulation Skills
Opportunity	Social	Social influences
	Physical	Environmental context & resources
Motivation	Reflective	Social/Professional Role & Identity Beliefs about capability Optimism Beliefs about consequences Intentions Goals
	Automatic	Social/Professional Role & Identity Optimism Reinforcement Emotion

Note: Adapted from Cane et al. (2012) [19].

Although the COM-B model and TDF are effective tools for guiding health interventions, their use in CC screening research in the rural Kilimanjaro region is limited. This study aims to fill this gap by applying the COM-B and TDF models to explore facilitators and barriers to CC screening among women and healthcare providers in the rural Kilimanjaro region and ultimately supporting the design of theory-based interventions that can effectively drive behavior change and increase CC screening uptake in the Kilimanjaro region [20].

## Methods

### Study design

This study used an explorative qualitative formative design with an inductive-deductive framework in accordance with Braun and Clark [21].

### Research question

*What are the facilitators and barriers to CC screening among women and healthcare providers?*

### Study settings

The study was conducted in the rural areas of the Kilimanjaro region, specifically in the Rombo and Moshi Rural districts, which are two of the seven districts in the region. The Kilimanjaro region has a total population of 1,861,934. Moshi Rural District counts 535,803 people, and Rombo District, 275,314 [22], chosen due to their higher prevalence of CC cases 36.4% and 15.2% respectively and lower CC screening rates compared to other districts (38% in Moshi and 46% in Rombo) [23]. Rombo District has 2 hospitals, 5 health centers, and 38 dispensaries, only 5 health facilities offering screening, while Moshi Rural District is home to 6 hospitals, 8 health centers, and 93 dispensaries with 6 facilities providing the CC screening service. In total, the region has 32 facilities equipped to provide CC screening [23].

### Participants

The participants included screened women, irrespective of duration of screening and test results, and non-screened women living in the community under study. The inclusion criteria were women aged 30–50 years (age recommended for screening according to the Tanzania CC Prevention and Control Guideline, 2023) [24]. Swahili speakers, and those who consented to participate. Exclusion criteria included women on CC treatment.

Healthcare providers (nurses and doctors) providing CC screening services were also included from both private and public screening facilities in Rombo and Moshi rural districts, regardless of years of experience and level of education.

### Selection of health facilities

Six CC screening facilities were purposively selected for the study based on a 1:2 ratio corresponding to the population sizes of their respective districts. This included 2 facilities from Rombo and 4 from Moshi Rural District. Among the 6 selected facilities, 3 were privately owned. The facilities selected from Moshi Rural District included TPC Hospital, and Himo OPD Health Centre (Government owned facilities), Kibosho Hospital and Kilema Hospital; while those from Rombo District included Huruma Hospital (private owned facilities) and Karume Health Centre Facilities were purposefully selected based on their geographical location, which ensures a diverse and representative sample population. The CC screening services provided by these facilities include visual inspection with acetic acid (VIA), follow-ups (tracking and reminding women of follow-up appointments and referrals for advanced care when necessary), pre- and post-screening education and counseling, and treatment of precancerous lesions.

### Selection of participants

Purposive sampling was used to recruit screened participants from the register provided by the Tanzanian Ministry of Health (MoH) at the health facilities. The register included information on serial number, client name, address, phone number, age, age at first sexual contact, HIV status, date of last menstrual period, screening results, use of cryotherapy, and any referrals for Loop Electrosurgical Excision Procedure (LEEP) or other treatments [25]. The phone numbers listed on the screening register were used to contact study participants. After verifying the inclusion criteria and providing verbal summary information about the project by phone, an invitation to participate in the study was extended. Moreover, similar approach was used to recruit non-screened women from the community [26]. Four community health workers (two in each district) were identified and acted as points of contact for non-screened women in the community. To ensure wide representation, the study recruited one woman per household; when more than one woman in a household was eligible, the study recruited the oldest (30–50 years).

The study recruited twelve healthcare providers (nurses and doctors) whose primary responsibility was CC screening, with two participants selected from each of the chosen health facilities using convenient sampling.

### Data collection

The Focus Group Discussion (FGD) guide was adopted from a previous study [27] without modifications, while the individual interview guide was adopted from two studies [28] and [29] and modified to fit the study. Both tools were based on the COM-B model (capability, opportunity, motivation) and the TDF domains, which include knowledge, social influences, beliefs, environmental context, resources, and social/professional role. The guides were translated into Swahili and back-translated by two independent experts to ensure accuracy [26].

Data were collected from 13^th^ March to 1 October 2024. In total, six FGDs were conducted, three from each district, which included a group of screened women, non-screened women, and a mixed group of screened and non-screened women to explore differing perspectives on facilitators and barriers to CC screening. Each group had 8–12 participants. The inclusion criteria of mixed groups aimed to encourage open dialogue and explore shared and differing experiences between screened and non-screened women, providing comprehensive insights into screening behaviors. Skilled facilitation ensured a safe, inclusive environment that supported mutual understanding and open participation. The discussions were moderated by the lead author, with the support of two trained assistants (a female nurse and a male socio-scientist). FGDs were conducted in accessible and comfortable venues and lasted up to 75 min. Information collected included participants’ CC and screening histories, screening test experiences, information-seeking behaviors, social support, and suggestions for improvement.

Semi-structured in-depth interviews (IDIs) with healthcare providers were conducted at their workplaces and lasted up to 40 min. Data saturation was reached at 10 interviews. However, all 12 participants were interviewed to confirm that no new information emerged. Information collected from the individual interviews included providers’ knowledge, role, perceptions, emotional experiences, peer dynamics, and the barriers and facilitators involved in supporting behavior change.

FGDs and IDIs were audio-recorded by a digital device, and notes were taken. Informed consent was obtained from all participants. Each participant completed a socio-demographic information form, which took a maximum of 5 min. Confidentiality was maintained through secured data handling practices.

FGDs captured community perspectives, while IDIs explored individual experiences, enabling data triangulation. Using separate methods helped balance differing knowledge and prevent dominance by healthcare providers.

### Data management and synthesis

All FGDs and IDIs were audio-recorded, transcribed verbatim, and translated into English by RFM and VWK. Thematic analysis with an inductive-deductive approach was used [21,30]. The consolidated criteria for reporting qualitative studies (COREQ) checklist was employed [31].

The transcripts were reviewed for accuracy and consistency with the audio recordings. The analysis included familiarization with the data, construction of a thematic framework based on the TDF domains, indexing, sorting data, and interpretation of data. An inductive-deductive coding framework was employed to avoid complications in the operationalization of the TDF domains [32]. Using an inductive approach, two researchers (RFM, VWK) generated themes for similar data clusters, and definitions for these themes were developed. For the deductive element, themes were categorized into domains based on a TDF-based coding manual, which provided clear guidelines for how the inductively generated themes would be categorized within the TDF framework.

Two researchers (RFM, VWK) independently read and coded two transcripts. Recurrent themes and coding were compared and discussed to find differences for consensus. Once the coding framework was finalized, the remaining interviews were coded. Each theme was then deductively mapped to the TDF domains and subsequently to the COM-B model. The TDF framework identified key domains such as knowledge, beliefs, and social influences, guiding the development of study tools, while the COM-B model was used to design questions that assess capability, opportunity, and motivation, focusing on factors influencing screening participation. Themes were mapped to the three COM-B components and their subthemes: Capability (psychological), Motivation (automatic, reflective), and Opportunity (social, physical), as well as the eight TDF domains: Knowledge, Social Influences, Emotion, Environmental Context and Resources, Social/Professional Role and Identity, Beliefs about Consequences, and Beliefs about Capabilities. The data were analyzed separately due to differences in the interview guides, thereby supporting methodological triangulation. Subsequently, the findings were integrated; however, they were distinguished through separate quotations and reported under shared thematic categories [33]. Common verbatim debates related to facilitators and barriers were selected and included in the analysis to illustrate the findings.

### Trustworthiness of the study data

The criteria for data saturation, credibility, dependability, transferability, and confirmability were implemented to reduce the likelihood of bias influencing the researchers’ interpretations during data analysis. These criteria are strongly advocated in qualitative research to enhance the reliability of study outcomes [26,34]. Credibility was strengthened by employing established research methodologies and collecting data directly from women and healthcare providers providing CC screening. Trustworthiness was upheld by selecting reliable participants and ensuring that the interviewer, who had no prior relationship with the participants, strictly followed the interview protocol. Additionally, thematic analysis guided the interpretation process, and two independent researchers reviewed the data separately. Their diverse perspectives promoted transparency and supported the study’s dependability and confirmability. Transferability was improved through detailed descriptions of the data collectors, data collection procedures, interview duration, and analysis methods. These details enable the findings to be applicable in other contexts [26,34].

## Results

### Characteristics of the study participants

Six FGDs involving 54 women were conducted, with 26 women screened and 28 not screened for CC. Women aged above 35 were 19 and 12 for screened and non-screened women, respectively. Additionally, 22 screened and 27 non-screened women had primary or secondary education, and nearly all participants were self-employed. IDIs were conducted among 12 healthcare providers, 9 of whom were female. Four of the 12 participants had more than 10 years of experience in providing CC screening. Nearly all participants (10) had a college or university education, and almost half of the healthcare providers or their spouses had not been screened for CC. Detailed information about the participants is shown in Table 3.

**Table 3.** Socio-demographic characteristics of study participants (*n* = 66).

Variable			Health Providers n (%)
	Screened women n (%)	Non-screened women n (%)	
**Sex**			
*Male*			3 (25)
*Female*	26 (100)	28 (100)	9 (75)
**Districts**			
*Rombo*	14 (53.9)	13 (46.4)	4 (33.3)
*Moshi District*	12 (46.1)	15 (53.6)	8 (66.7)
**Data Collection Method**			
*FGD*	26 (100)	28 (100)	
*IDI*			12 (100)
**Age**			
*<35 years old* *≥35 years old*	7 (26.9) 19 (73.1)	16 (57.1) 12 (42.9)	
**Years: experience in providing CC screening services**			
*<10 years of experience*			8 (66.7)
*>10 years of experience*			4 (33. 3)
**Educational levels**			
*Primary Education*	18 (69.2)	18 (64.3)	–
*Secondary*	4 (15.4)	9 (32.1)	2 (16.7)
*College and University*	4 (15.4)	1 (3.6)	10 (83.3)
**Health Facility**			
*Hospital*			8 (66.7)
*Health Centre*			4 (33.3)
**Employment status**			
*Self employed*	23 (88.5)	27 (96.4)	–
*Employed*	3 (11.5)	1 (3.6)	12 (100)
**Occupation**			
*Nurses*			8 (66.7)
*Doctors*			4 (33.3)
**Screened for CC**			
*Yes*	26 (48.2)		7 (58.4)
*No*		28 (51.8)	5 (33.3)

### Results of the data analysis

Four main themes emerged from this study, each with sub-themes: 1. Knowledge of CC and screening (disparities in knowledge between screened and non-screened women; healthcare providers’ knowledge and training needs; age and parity as knowledge mediators); 2. Power of Social Influence (social facilitation; social barriers); 3. Emotional and structural influences on CC screening (emotional barriers and coping; structural and logistical barriers); and 4. Enhancing CC screening uptake (targeted community education and outreach; improving service accessibility). The results are presented for each main theme and sub-theme, corresponding to the COM-B constructs and TDF domain(s), featuring illustrative quotes throughout the main text.

#### Knowledge of CC and screening

This theme was mapped to the TDF domain of knowledge as participants exhibited varying levels of awareness regarding risks, prevention, and screening services. Within the COM-B model, this corresponds to psychological capability, highlighting the role of cognitive understanding in enabling screening behavior. Three sub-themes were identified: disparities in knowledge between screened and non-screened women, healthcare providers’ knowledge and training needs, and age and parity as knowledge mediators.

##### Disparities in knowledge between screened and non-screened women

Non-screened women did not know that CC is a serious health threat with a high mortality rate; they believed that screening was only necessary if symptoms or pain were present, not realizing that the disease can progress silently in its early stages. One of the non-screened participants reported that;

… Many people don’t know what CC is. They just hear the name, but if you ask them to explain, they won’t be able. There is very little knowledge, and that is a big problem”(FGD5-P2).

While the majority of screened participants demonstrated greater knowledge of CC and recognized it as a serious disease, which served as a motivation for undergoing screening. A screened woman explained,

… CC develops without noticeable symptoms, making you feel healthy even if it’s progressing. Over time, it may reach an advanced stage, and only then you will start experiencing the pain. By the time you seek medical help, the condition may have become severe, and you could be in stage 4 (FGD 2-P1).

However, nearly all of the screened women had limited knowledge of the causes of CC.

Non-screened participants also showed limited knowledge of CC screening, including the HPV self-sampling tests. This was often attributed to insufficient education and a lack of accessible information. Several expressed a desire for more comprehensive education on the benefits of screening and the risks associated with not being screened:

… I wish to know the benefits of screening or the risks of not screening. (FGD5-P2)

Furthermore, the study revealed confusion about which women are most at risk of developing CC and highlighted differing perceptions of screening between screened and non-screened participants. One non-screened participant said;

… I have never been screened because I have never had any problems. (FGD4-P9)

##### Healthcare providers’ knowledge and training needs

The study found that healthcare providers were knowledgeable about CC screening, risk factors, eligibility, treatment, and referral procedures, which they gained through formal and on-the-job training. While expected, confirming their knowledge ensures accurate screening services and highlights potential practice inconsistencies. Healthcare providers recommended continuous education and refresher courses to enhance their skills;

… As healthcare providers, we need continuous updates and refresher training to stay competent. The more we practice and learn, the better we can guide women through the screening process (IDI 10)

Age and parity as knowledge mediators There was a clear age-related difference, with most screened women being over 35 years old and most non-screened women under 35. This may be due to older women having more knowledge on CC, greater access to information, and more frequent contact with healthcare services. Parity could also play a role, as women with more children are more likely to use maternal health services where screening is promoted.

#### Power of social influence

This theme describes how social relationships and community dynamics influence women’s CC screening decisions. It was mapped to the social influences domain of the TDF, reflecting the role of family, peers, and community norms in shaping behavior. Within the COM-B model, these factors represent social opportunity, as they create external conditions that facilitate or hinder screening uptake. Two sub-themes were identified: social facilitation, and social barriers.

##### Social facilitation: family, peers, community leaders, and healthcare providers

We found that support from healthcare professionals, friends, family, religious leaders, and community leaders influenced women’s decisions to undergo CC screening. Screened participants reported that awareness campaigns at the community level have motivated them to seek screening; non-screened participants were still affected by misinformation and discouragement, suggesting that these campaigns did not effectively reach all community members. A screened woman said;

… Community initiatives and awareness campaigns were key in changing my view on cervical cancer screening and encouraged me to take it seriously (FGD5-P8)

The study also highlighted the crucial role of healthcare providers (HCPs) in supporting women through education, addressing misconceptions, and offering assistance, such as follow-up calls and transportation. Participants acknowledged that HCPs’ involvement, along with community awareness campaigns and seminars, helps create social opportunities to spread information and motivate more women to participate in screening, thus contributing to family and community well-being.

Mass screenings are helpful, so we should continue educating and advertising, perhaps in churches, mosques, and markets (IDI 01).

##### Social barriers: misinformation, rumors, and uneven reach of campaigns

The findings highlight that misinformation, fear-based rumors, and inadequate dissemination of accurate health information can reduce community participation in screening programs by fostering misconceptions about the safety of screening procedures. One of the non-screened participant reported that;

… . when we are in our groups, there are rumors here and there, like, hey, don’t dare go for screening; if they touch it, you will die (FGD5-P9).

#### Emotional and structural influences on CC screening

This theme captures psychological and systemic determinants of women’s participation in CC screening. It maps to the TDF domains of Emotions and Environmental Context and Resources, reflecting affective barriers (fear, embarrassment, anxiety) and systemic constraints (access, cost, service availability). Within the COM-B model, these correspond to automatic motivation and physical opportunity, respectively, highlighting how emotional responses and external conditions jointly influence screening behavior. Two sub-themes were identified: emotional barriers and coping, and structural and logistical barriers.

##### Emotional barriers and coping

Under the domain of *Emotions* and *Automatic Motivation*, both screened and non-screened women expressed fears related to pain, discomfort, and the possibility of receiving positive results. Screened women attributed their anxieties to personal experiences with the screening procedure, whereas community narratives and misconceptions influenced non-screened women. Despite these concerns, some screened women felt confident in coping with the procedure, recognizing the temporary nature of the pain and the seriousness of the disease. They also encouraged others to get screened;

I understand this disease (meaning ‘CC’) is dangerous, and the pain experienced from speculum insertion is temporary. (FGD1-P3)

Screened participants highlighted relaxation techniques that helped manage stress, emphasizing the importance of psychological preparation and support;

Learning about the screening process and having supportive guidance has made me more skilled in coping with the procedure. I now feel more confident in managing the screening experience. (FGD3-P9)

##### Structural and logistical barriers

HCPs found fulfillment when women accepted screening but noted that persistent myths and misconceptions continued to act as barriers to acceptance. Nevertheless, they remained committed to educating and motivating women, even when encouraging screening in the absence of symptoms proved challenging:

I feel very motivated when I see that my counseling helps women take charge of their health. (IDI 03)

From the perspective of *environmental context and resources* and *physical opportunity*, several structural and systemic barriers were identified. Although screened women generally viewed access to services positively, they emphasized the need for ongoing education and greater awareness. Reported barriers included forgetfulness, discomfort with the speculum, limited availability of screening facilities, particularly in rural areas, and a shortage of trained healthcare personnel. These limitations hindered follow-up care and strained providers’ ability to offer adequate support and education:

One of the biggest challenges is the shortage of healthcare providers. If we had more trained personnel, we could screen more women and provide better follow-up care. (IDI 11)

#### Enhancing CC screening uptake

This theme captures strategies to enhance women’s participation in CC screening. It maps to TDF domains of Environmental Context and Resources (improving service accessibility), Social/Professional Role and Identity (influence of healthcare providers and community leaders), Beliefs about Consequences (awareness of screening benefits), and Beliefs about Capabilities (confidence to participate). Within the COM-B model, these factors align with reflective motivation, as they shape deliberate evaluations and intentions toward screening uptake. Two sub-themes were identified: targeted community education and outreach, and improving service accessibility.

##### Targeted community education and outreach

The findings highlight that targeted community education and the use of multiple communication channels can improve awareness, information dissemination, and participation in screening programs by enhancing community reach and engagement. A screened participants reported that;

… disseminating information or raising awareness within the community should not rely on just one method; a combination of methods is needed to reach the community effectively, such as through churches, mosques, organizing meetings in villages, broadcasting through media outlets, and using fliers for awareness. (FGD2-R8)

##### Improving service accessibility

Participants suggested using community meetings, religious gatherings, media, and fliers to spread information. While HCPs highlighted the challenge of reaching women outside hospitals, they recommended outreach in villages and markets to increase awareness and participation;

Going to the villages to provide education on CC because many community members are unaware of these services and might never come to the hospital on their own. (IDI 06)

Participants stressed the importance of involving men in the process, as some husbands may discourage their wives from getting screened. Additionally, bringing screening services closer to communities, offering tests during sensitization campaigns, and providing clear results and treatment information were recommended to improve accessibility and encourage more women to participate.

## Discussion

Our study aimed to explore the facilitators and barriers to CC screening among women and healthcare providers. Four key themes, each comprising of sub-themes emerged from the analysis. The findings revealed that major barriers included limited knowledge, misconceptions, and emotional fears related to pain and diagnostic outcomes. In contrast, key facilitators included access to accurate information, supportive interpersonal relationships, and active community engagement.

### Knowledge of CC and screening

Reflecting the sub-themes of disparities in knowledge and healthcare providers’ knowledge and training needs, we found that non-screened women in the rural Kilimanjaro region had limited knowledge about CC, its causes, and risk factors, often believing that screening was only necessary when symptoms or pain were present. Contributing factors included insufficient education, inadequate communication from healthcare providers, and widespread misconceptions. These findings are similar with studies conducted in Kenya in 2022 [8], sub-Saharan Africa in 2020 [12], Tanzania in 2021 [25] and South Africa in 2017 [35], which also identified limited knowledge about CC and deficiencies in provider’s education as significant barriers. Although screened women understood the seriousness of CC, their knowledge of its cause was limited, possibly due to health education focusing more on the screening process than on HPV. This gap may be exacerbated by shortages of healthcare providers. In contrast, a study conducted in Nigeria reported limited knowledge of HPV [36]. Furthermore, non-screened women demonstrated limited knowledge of CC screening. This knowledge gaps among non-screened women increase their risks, highlighting the need for targeted education to support informed screening choices. Engaging community leaders and community health workers can boost screening rates and encourage behavior change. The knowledge difference between screened and non-screened women is likely the result of a combination of health system exposure [9], and social influence. These findings emphasize the importance of using the screening process as a platform for education, awareness, and expanding outreach efforts to reach women who do not typically access health services.

### Power of social influence

Consistent with the sub-themes of social facilitation and social barriers, the present finding highlights the power of social support on informational (sharing accurate details about CC, screening, and its benefits), emotional (encouragement and reassurance), and practical roles (e.g. assistance with logistics like transportation) in facilitating CC screening uptake. Support from healthcare providers, family, friends, and religious leaders significantly influenced women’s screening decisions. Screened women reported that encouragement from family and personal interactions with healthcare professionals positively shaped their attitudes toward CC screening [37]. In contrast, non-screened women often encountered negative social influences, including misinformation and discouragement, which impeded their engagement with screening services. Additionally, a disparity was observed between positive social influences and actual access to screening opportunities, particularly among women in underserved areas [28]. These highlight the need for more inclusive and targeted approaches to ensure that no subgroup is left behind. Intervention should integrate education, emotional support, and community-based outreach. In a study conducted in Zimbabwe 2017 [14], further emphasized the importance of educating women, particularly those from traditional churches, to encourage CC screening. Enhancing social opportunities through targeted communication strategies and environmental planning may also increase CC screening participation, particularly among women facing social and logistical barriers [38].

### Emotional and structural influences on CC screening

Aligned with the sub-themes of emotional barriers and coping, and structural and logistical barriers, this study highlights the combined impact of emotional and structural factors on CC screening, framed through the TDF and COM-B models. Women experienced emotional barriers, particularly fears of pain, diagnosis, and discomfort associated with the speculum [37]. The origins of these fears varied, stemming from either personal experiences or community-driven misconceptions. In contrast, studies by [39] and [40] found that women frequently held negative perceptions of CC screening, often reporting feelings of fear and embarrassment.

Some screened women developed coping strategies, which boosted their confidence and willingness to encourage others to participate in screening. These results demonstrate the need for psychological support, education, and training to reduce emotional barriers and enhance women’s confidence. These results are similar to a study done in South Africa that supports community education programs and mass media campaigns to spread information about CC and address negative community perceptions [35].

Healthcare providers played a key role in motivating women but faced challenges in promoting screening among asymptomatic individuals, particularly outside clinical settings. Addressing these challenges requires broader community outreach to strengthen both psychological capability and practical opportunity for CC screening participation. In contrast, a 2017 study conducted in Kenya reported that women who underwent screening did so based on recommendations from their healthcare providers [37].

Structurally, the study highlights speculum discomfort, limited-service availability, and shortages of healthcare providers, especially in rural areas, as significant barriers. These findings emphasize the need for theory-informed interventions that address both individual and systemic obstacles to improve screening uptake, including education, peer support, and service delivery enhancements.

### Enhancing CC screening uptake

Reflecting the sub-themes of targeted community education and outreach, and improving service accessibility, the present study emphasized the need for improved access to education and services. Utilizing diverse outreach platforms where most women are found, such as religious gatherings, markets, community meetings, women’s groups, and social media, can raise awareness and improve knowledge, especially in rural communities [41,42]. Participants indicated that understanding CC would motivate screening and recommended mobile clinics and the use of community health workers to address distance barriers [29]. Involving community leaders in advocacy enhances trust, reduces stigma, and promotes culturally accepted health initiatives. This is supported by [43], who reported that community-based educational programs can enhance awareness of CC and screening, foster positive attitudes towards the benefits of screening, and help reduce perceived barriers to accessing it. Additionally, engaging men in CC screening promotion is crucial, as their support influences women’s health decisions; however, the majority of men reported having limited information on CC screening [38]. Findings from this present study also supported the HPV self-sampling test, highlighting its comfort and potential to increase screening if made affordable and accessible. A comprehensive approach that combines education, community involvement, service accessibility, male engagement, and HPV self-sampling testing was reported to enhance screening uptake, reduce mortality, and improve health equity and outcomes in rural populations.

### Strengths and limitations

This study demonstrated several qualitative strengths that contributed to the trustworthiness and rigor of its findings. It employed the four widely recognized criteria of qualitative trustworthiness: credibility, dependability, transferability, and confirmability [34,44]. Data were collected directly from women and healthcare providers actively involved in CC screening, ensuring relevance and capturing firsthand experiences. The interviewer-maintained neutrality, having no prior relationship with participants and strictly adhering to the interview protocol to minimize potential bias. Additionally, thematic analysis was used to guide interpretation, and two independent researchers reviewed the data separately, thereby strengthening dependability and reducing individual bias. Detailed descriptions of the data collection process, interview procedures, and analytical methods further enhanced transferability, allowing the findings to be considered in other similar contexts.

The study faced limitations. The participant scope was limited to women and healthcare providers with a disproportionate number of nurses compared to doctors, reflecting the staffing structure of the clinics, but potentially skewing the findings toward a nursing viewpoint, excluding administrative personnel perspectives. Despite measures to reduce bias, the researchers’ interpretations could still have been influenced by their assumptions.

## Conclusion

CC screening uptake can be improved by addressing the barriers and facilitators identified across the four themes and their sub-themes. These include expanding knowledge and provide training (disparities in knowledge; healthcare providers’ training needs), strengthening social support and community education (social facilitation; targeted outreach), addressing emotional barriers through psychological preparation and peer support (emotional barriers and coping), and resolving structural gaps in service access and workforce (structural and logistical barriers).

## Supplementary Material

COREQ Checklist Revised.docx

## Data Availability

The data presented in this study are available on request from the corresponding author.

## Pre-print

This manuscript is posted to a preprint server. Link to the pre-print:

https://www.medrxiv.org/content/10.1101/2025.8 January 25332778v1.full-text.
