# Supplementary material for: Perspectives of women and healthcare providers on facilitators and barriers to cervical cancer screening in the rural Kilimanjaro region Tanzania: a qualitative study
Source: Glob Health Action. 2026 Sep 28;19(1):2735552. doi: 10.1080/16549716.2026.2735552 (PMC13629785; doi:10.1080/16549716.2026.2735552)
Supplement: Supplementary file 1 [file ZGHA_A_2735552_SM7366.docx]

**Consolidated criteria for reporting qualitative studies (COREQ): 32-item checklist**

| **No** | **Item** | **Guide questions/description** | **Response** |
| --- | --- | --- | --- |
| Domain 1: Research team and reflexivity | | | |
| Personal Characteristics | |  |  |
| 1 | Interviewer/facilitator | Which author/s conducted the interview or focus group? | Rogathe F. Machange  Christina C. Mtuya  Victor W. Katiti |
| 2 | Credentials | What were the researcher’s credentials? E.g. PhD, MD | MSc, PhD Student, MD, PhD |
| 3 | Occupation | What was their occupation at the time of the study? | Researchers, Academicians and Healthcare Providers |
| 4 | Gender | Was the researcher male or female? | Female and Male |
| 5 | Experience and training | What experience or training did the researcher have? | GCP  Experienced in community and hospital based studies in Qualitative approach |
| Relationship with participants | | | |
| 6 | Relationship established | Was a relationship established prior to study commencement? | NO |
| 7 | Participant knowledge of the interviewer | What did the participants know about the researcher? e.g. personal goals, reasons for doing the research | The interviewer explained goals and reasons of study prior to the interview for participants to be informed. |
| 8 | Interviewer characteristics | What characteristics were reported about the interviewer/facilitator? e.g. Bias, assumptions, reasons and interests in the research topic | NONE |
| Domain 2: study design | | | |
| Theoretical framework | | | |
| 9 | Methodological orientation and Theory | What methodological orientation was stated to underpin the study? e.g. grounded theory, discourse analysis, ethnography, phenomenology, content analysis | An exploratory |
| Participant selection | | | |
| 10 | Sampling | How were participants selected? e.g. purposive, convenience, consecutive, snowball | Purposive |
| 11 | Method of approach | How were participants approached? e.g. face-to-face, telephone, mail, email | Face-to-face |
| 12 | Sample size | How many participants were in the study? | 66 (54 Women and 12 Healthcare providers) |
| 13 | Non-participation | How many people refused to participate or dropped out? Reasons? | NONE |
| Setting | | | |
| 14 | Setting of data collection | Where was the data collected? e.g. home, clinic, workplace | Community and Clinics in healthcare facilities |
| 15 | Presence of non-participants | Was anyone else present besides the participants and researchers? | NONE |
| 16 | Description of sample | What are the important characteristics of the sample? e.g. demographic data, date | demographic data |
| Data collection | | | |
| 17 | Interview guide | Were questions, prompts, guides provided by the authors? Was it pilot tested? | Yes |
| 18 | Repeat interviews | Were repeat interviews carried out? If yes, how many? | Not Applicable |
| 19 | Audio/visual recording | Did the research use audio or visual recording to collect the data? | Audio recording used |
| 20 | Field notes | Were field notes made during and/or after the interview or focus group? | Field notes were taken |
| 21 | Duration | What was the duration of the interviews or focus group? | Lasted for 40- 75 minutes |
| 22 | Data saturation | Was data saturation discussed? | Yes |
| 23 | Transcripts returned | Were transcripts returned to participants for comment and/or correction? | No |
| Domain 3: analysis and findings | | | |
| Data analysis | | | |
| 24 | Number of data coders | How many data coders coded the data? | Two |
| 25 | Description of the coding tree | Did authors provide a description of the coding tree? | YES |
| 26 | Derivation of themes | Were themes identified in advance or derived from the data? | Derived from the data |
| 27 | Software | What software, if applicable, was used to manage the data? | QDA Miner Lite v2.0.8 |
| 28 | Participant checking | Did participants provide feedback on the findings? | NO |
| Reporting | | | |
| 29 | Quotations presented | Were participant quotations presented to illustrate the themes / findings? Was each  Quotation identified? e.g. participant number | YES |
| 30 | Data and findings consistent | Was there consistency between the data presented and the findings? | YES |
| 31 | Clarity of major themes | Were major themes clearly presented in the findings? | YES |
| 32 | Clarity of minor themes | Is there a description of diverse cases or discussion of minor themes? | YES |
